# Mood dynamics in bipolar disorder

**DOI:** 10.1186/s40345-014-0011-z

**Published:** 2014-09-03

**Authors:** Paul J Moore, Max A Little, Patrick E McSharry, Guy M Goodwin, John R Geddes

**Affiliations:** Mathematical Institute, University of Oxford, Woodstock Road, Oxford, OX2 6GG UK; Aston University, Birmingham, B4 7ET UK; School of Geography and the Environment, University of Oxford, South Parks Road, Oxford, OX1 3QY UK; Department of Psychiatry, University of Oxford, Oxford, OX3 7JX UK

**Keywords:** Bipolar disorder, Mood dynamics, Time series analysis, Public healthcare

## Abstract

The nature of mood variation in bipolar disorder has been the subject of relatively little research because detailed time series data has been difficult to obtain until recently. However some papers have addressed the subject and claimed the presence of deterministic chaos and of stochastic nonlinear dynamics. This study uses mood data collected from eight outpatients using a telemonitoring system. The nature of mood dynamics in bipolar disorder is investigated using surrogate data techniques and nonlinear forecasting. For the surrogate data analysis, forecast error and time reversal asymmetry statistics are used. The original time series cannot be distinguished from their linear surrogates when using nonlinear test statistics, nor is there an improvement in forecast error for nonlinear over linear forecasting methods. Nonlinear sample forecasting methods have no advantage over linear methods in out-of-sample forecasting for time series sampled on a weekly basis. These results can mean that either the original series have linear dynamics, the test statistics for distinguishing linear from nonlinear behaviour do not have the power to detect the kind of nonlinearity present, or the process is nonlinear but the sampling is inadequate to represent the dynamics. We suggest that further studies should apply similar techniques to more frequently sampled data.

**Electronic supplementary material:**

The online version of this article (doi:10.1186/s40345-014-0011-z) contains supplementary material, which is available to authorized users.

## Background

The increase in digital communication and measurement has generated new medical data sets for analysis. These have in turn provided the opportunity to analyse biosignals which have hitherto been hard to measure. This study uses a new data set of depression time series recorded from outpatients who have bipolar disorder. Past work has claimed both deterministic chaos and stochastic nonlinearity for mood in bipolar disorder (Gottschalk et al. 1995; Bonsall et al. 2012). We address the latter claim directly and search for evidence of nonlinearity in eight selected time series.

### Previous work

Until recently, most analyses of mood in bipolar disorder have been qualitative. Detailed quantitative data has been difficult to collect: the individuals under study are likely to be outpatients, their general functioning may be variable and heterogeneous across the cohort. The challenges involved in collecting mood data from patients with bipolar disorder has influenced the kinds of study that have been published. Some investigators have proposed theoretical models which do not use observational data directly. Daugherty et al. (2009) proposed a nonlinear oscillator model and used a dynamical systems approach to describe mood changes in bipolar disorder. Steinacher and Wright (2013) also used a dynamical systems approach and used it to model the regulation of behavioural activation. Along similar lines, Buckjohn et al. examined the dynamics of two coupled nonlinear oscillators and related these to interpersonal interactions involving patients with bipolar disorder. Frank (2013) used a nonlinear oscillator approach and made a connection between this and biochemical modeling of the disorder. Where data has been analysed, then either detailed data has been taken from a small number of patients (Gottschalk et al. 1995; Wehr and Goodwin 1979) or more general data from a larger number (Judd 2002; Judd et al. 2003). The article by Wehr and Goodwin (1979) used twice daily mood ratings for five patients. Judd et al. (2002; 2003) measured patients’ mood using the proportion of weeks in the year when symptoms are present. This kind of measurement lacks the frequency and the resolution for time series analysis.

The ratings from questionnaires (Pincus 2003) have commonly been summarized using mean and standard deviation although other measures have been used. Pincus (1991) introduced *approximate entropy* as a measure of time series regularity or predictability. It was applied to both mood data generally (Yer-agani et al. 2003) and to mood in bipolar disorder (Glenn et al. 2006), where 60 days of mood data from 45 patients was used for the analysis. It was also applied in another study to distinguish between the pre-episodic and other states (Bauer et al. 2011). A summary of papers which report a temporal bipolar mood analysis is given in Table 1.

**Table 1:** **Analyses of mood dynamics in bipolar disorder**

**Authors**	**Subjects**	**Scale**	**Mood metrics**
Wehr and Goodwin (1979)	BP1/2 (*n*=5)	Bunney-Hamburg	None
Gottschalk et al. (1995)	BP (*n*=7)	Analogue	Linear, nonlinear
Judd (2002)	BP1 (*n*=146)	PSR scales	% of weeks at level
Judd et al. (2003)	BP2 (*n*=86)	PSR scales	% of weeks at level
Glenn et al. (2006)	BP1 (*n*=45)	Analogue	Approx entropy
Bonsall et al. (2012)	BP1/2 (*n*=23)	QIDS-SR	Linear, nonlinear
Moore et al. (2012)	BP1/2 (*n*=100)	QIDS-SR	Linear, nonlinear
Moore et al. (2013)	BP1/2 (*n*=100)	QIDS-SR	Linear, nonlinear

### Claim of chaotic dynamics

Gottschalk et al. (1995) analysed daily mood records from 7 patients with bipolar disorders and 28 normal controls. The 7 patients with bipolar disorders all had a rapid cycling course; that is, they had all experienced at least 4 affective episodes in the previous 12 months. Patients kept mood records on a daily basis (controls on a twice-daily basis) by marking mood on an analogue scale each evening to reflect average mood over the previous 24 h. The selected participants kept records for a period of 1 to 2.5 years.

Out of the seven patients, six had correlation dimensions which converged at a value less than five, while for controls, the convergence occurred no lower than eight. Equivalent surrogate time series did not show convergence with dimension. From these results, the authors inferred the presence of chaotic dynamics in the time series from patients with bipolar disorder. They noted the unreliability of correlation dimension as an indicator of chaos, but adduced the results from time plots, spectral analysis and phase-space reconstruction to demonstrate the difference from controls. The claim was challenged by Krystal et al. (1998) who pointed out that the power-law behaviour is not consistent with chaotic dynamics. In their reply (Gottschalk et al. 1998), Gottschalk et al. commented that the spectra could equally be fitted by an exponential model. The authors did not investigate the Lyapunov spectrum, which can provide evidence of chaotic dynamics. Their claim of deterministic chaos rested mainly on the convergence of correlation dimension and, as they acknowledged, this is not definitive (McSharry 2005). Their change of model for spectral decay weakened the original claims further - the evidence does not support nor deny it.

### Nonlinear time series

Bonsall and his co-authors (Bonsall et al. 2012) applied time series methods to self-rated depression data from patients with bipolar disorder. The focus of their study was mood stability: they noted that while treatment has often focused on understanding the more disruptive aspects of the disorder such as episodes of mania, the chronic week-to-week mood instability experienced by some patients is a major source of morbidity. The aim of their study was to use a time series approach to describe mood variability in two sets of patients, stable and unstable, whose members are classified by clinical judgement. The time series data were obtained from the Department of Psychiatry in Oxford and was from 23 patients monitored over a period of up to 220 weeks^a^. Patients were divided into two sets of stable and unstable mood based on a psychiatric evaluation of an initial 6-month period of mood score data. Their classification into two groups was made on the basis of mood score charts and non-parametric statistical analysis which is described further in (Holmes et al. 2011). The depression data for each group was then analysed using descriptive statistics, missing value analysis (including the attrition rate) and time series analysis.

The time series analysis was based on applying standard and threshold autoregressive models of order 1 and 2 to the depression data for each patient. The authors concluded that the existence of nonlinear mood variability suggested a range of underlying deterministic patterns. They cited the claims of Gottschalk et al. (1995), though not the reply to them (Krystal 1998). They suggested that the difference between the two models could be used to determine whether a patient would occupy a stable or unstable clinical course during their illness and that the ability to characterize mood variability might lead to treatment innovation.

### Discussion

This study has a well-founded motivation. There is evidence that symptoms of bipolar disorder fluctuate over time and it is common for patients to experience problems with mood between episodes (Judd 2002). In approaching time series with unknown dynamics, the use of an autoregressive model is an obvious starting point. However, there are signs that the model fit is poor in this case. The distribution of RMSE values for the stable patients is reported to have a median of 5.7 (0.21 when normalised by the maximum scale value) and the distribution for the unstable patients, a median of 4.1 (0.15 normalised) (Bonsall et al. 2012, Data supplement). Further, we note that these are in-sample errors rather than expected prediction errors estimated by out-of-sample forecasting. As such, they are rather high compared with the reported standard deviations (3.4 for the stable group and 6.5 for the unstable group (Bonsall et al. 2012)) suggesting that the unconditional mean might be a better model for the stable group.

The reason for the poor model fit is not clear. A contributing factor might be that the time series are non-stationary: visual inspection of Figures four and five in (Bonsall et al. 2012) shows a variation in mean for some of the time plots. To mitigate non-stationarity, a common technique is to difference the time series, as in ARIMA modelling, or to use a technique that has the equivalent effect, such as simple exponential smoothing.

## Methods

In the present study, we search for evidence of nonlinearity using linear surrogates, then compare the expected prediction error of linear and nonlinear forecasting methods on eight selected patients. Time series data was collected as part of the *OXTEXT* (http://oxtext.psych.ox.ac.uk/) programme which investigates the potential benefits of mood self-monitoring for people with bipolar disorder. OXTEXT uses the *True Colours* (https://truecolours.nhs.uk/www/) self-management system for mood monitoring which was initially developed to monitor outpatients with bipolar disorder. Each week, patients complete a questionnaire and return the results as a digit sequence by text message or email. The resulting time series of mood ratings are visualised as color-coded graphs for use at an outpatient appointment. This information is used both by clinicians to select appropriate interventions and by the patients themselves for management of their condition. The Oxford mood monitoring system has generated a large database of mood time series which has been used for studying the longitudinal course of bipolar disorder (Bopp et al. 2010) and for nonlinear approaches to characterising mood by Bonsall et al. (2012). The data in the current study used the same telemonitoring techniques and the same rating scales as (Bonsall et al. 2012), but the studies are otherwise independent.

The work reported here has been performed in accordance with Declaration of Helsinki of 1975, as revised in 2004, and was approved by the local Research Ethics Committee ref: 10/H0604/13.

### The data

**Figure 1:**
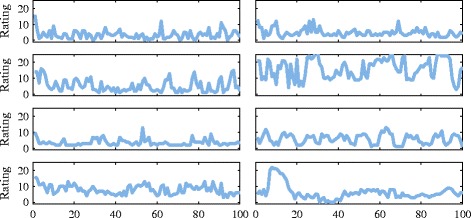
**Time series from patients used in the study, showing the first 100 points.** Each plot shows depression ratings sampled on an approximately weekly basis. The maximum possible score on the rating scale for depression (QIDS) is 27.

The data used in this study is derived from eight patients with bipolar disorder whose mood was monitored over a period of 5 years. The mood data is returned approximately each week and comprises answers to standard self-rating questionnaires for depression and mania. We restrict the investigation to the depression data which is more amenable to analysis than mania: for some patients, the mania scores are at or near zero for the period of monitoring. The rating scale used for depression is the *Quick Inventory of Depressive Symptomatology - Self Report (QIDS-SR *_16_*)* (Rush et al. 2000) which covers nine symptom domains for depression (DSM-IV-TR) (American Psychiatric Association 2000). This scale has been evaluated for psychometric properties and found to have high validity (Rush 2003). Each domain can contribute up to 3 points giving a total possible score of 27 on the scale. Details of the selection process are given in Additional file 1: Section I and plots of the selected time series are shown in Figure 1.

#### Descriptive statistics

The statistical qualities of the 8 patients in the study are shown in Table 2, which includes a comparison with a larger set of 93 patients from which the subset is selected. The patients are selected using the following criteria: (1) a minimum time series length of 100 ratings (only the first 100 ratings are used in the analysis), (2) fewer than 5 missing ratings in the time series and (3) stationarity, measured by comparing the rating distribution between the first and second parts of the time series. Diagnostic subtypes (bipolar I, bipolar II, bipolar NOS) are available for only some of the participants. The depression for each patient is first summarised by its mean, and the median/interquartile range for the mean is shown for each set of patients.

**Table 2:** **Age, length and mean depression showing median ± interquartile range**

**Set (** ***n*** **)**	**Sex F/M**	**BP I/II**	**Age**	**Length**	**Depression**
			**(years)**	**(points)**	**(QIDS)**
A (93)	60/33	41/22	45±20	136±161	6.4±5.3
B (8)	6/2	4/2	48±28	181±67	5.1±2.5

### Surrogate data analysis

The purpose in this section is to examine evidence of nonlinear dynamics in the mood time series. As a first test, the data are examined for correlation structure: if a time series has no serial correlation, then genuine forecasts cannot be made from it. An empirical approach to this analysis is the method of *surrogate data* (Lu 2004; Kantz and Schreiber 2004). To test for correlation structure, we permute the original series several times to obtain a surrogate set with series having the same amplitude but from a random process. A test statistic is then applied to the original and the surrogates and the results displayed graphically to see if there is a difference. For the null hypothesis of white noise, we use the autocorrelation at varying lags as a test statistic.

Next, we consider the null hypothesis of a linear stochastic model with Gaussian inputs. If this cannot be not rejected, then there is a question over the use of more complex, nonlinear models for forecasting. For this analysis, the surrogate data must be correlated random numbers with the same power spectrum as the original data. This is a property of data which has the same amplitude as the original data but in different phases. Amplitude-adjusted Fourier transform (AAFT) surrogates (Kantz and Schreiber 2004) have a slightly different power spectrum from the original series because the original untransformed linear process has to be estimated. To make the surrogates match the original spectrum more closely, we use corrected AAFT (CAAFT) surrogates (Kugiumtzis 2000).

## Results

**Figure 2:**
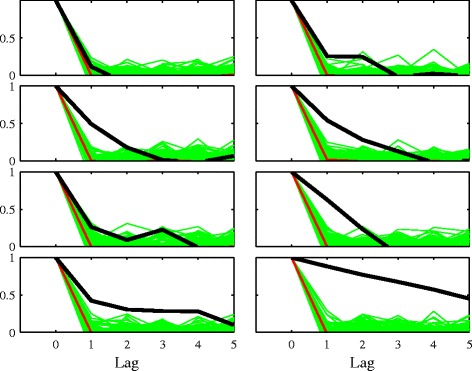
**Sample autocorrelation statistic for original eight time series and their shuffle surrogates.** For each of the eight time series, surrogates are created by randomly permuting the original time series. The sample autocorrelation function up to a lag of 6 is then shown for the surrogates, and that for the original series is shown in a dark line. The thin red line shows the median of the surrogates. The top left plot shows a time series that is indistinguishable from white noise. The other time series show serial correlation.

We first examine the sample autocorrelation function for the eight time series and compare it with that for surrogates which have the same distribution. Figure 2 shows the results, with the sample acf shown for lags 1 to 6. Seven of the eight time series are distinct from their shuffle surrogates, showing that they have some serial correlation. The time series in the top left panel of Figure 2 cannot be distinguished from its permutations and thus is unsuitable for the purposes of forecasting. We keep this time series in the experimental set to provide a contrast to the other time series.

### Testing for nonlinearity

**Figure 3:**
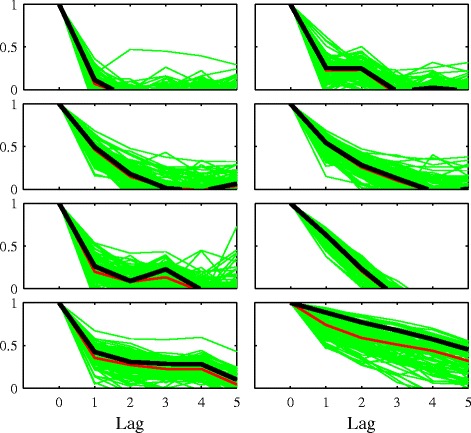
**Sample autocorrelation statistic for the original eight time series and their CAAFT surrogates.** In most cases, the original time series is typical among the surrogates with the exception of the plot on the lower right which shows a downward bias in the surrogates.

Figure 3 shows the autocorrelation of CAAFT surrogates compared with the original eight time series used in this study. The MATLAB software associated with (Kugiumtzis 2000) is used to generate the CAAFT surrogates. It can be seen that they reproduce the original autocorrelation well in most cases although those in the lower right panel are biased downwards.

**Figure 4:**
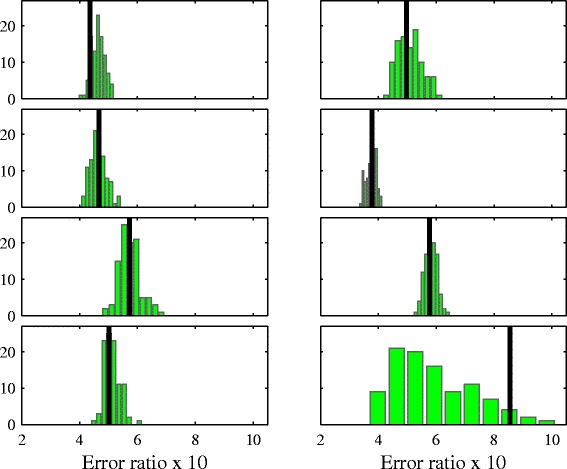
**In-sample error ratio for nonlinear vs linear modelling.** The original time series is represented as a vertical line and the errors for the surrogates are shown as a histogram. The panel on the bottom-right shows that the original series is relatively better forecast by the linear than the nonlinear method. This anomaly is caused by the surrogates having an artificially lower average correlation, shown in the equivalent panel in Figure 3.

We next compare the ratio of linear vs. nonlinear in-sample forecast error for the original and surrogate time series. If there is nonlinearity present, we would expect the nonlinear method to show an improvement over the linear method. The linear method used is persistence, and the nonlinear method is a zero-order nearest-neighbor method which is described in Additional file 1: Section II and implemented in the *TISEAN* function lzo-run (Hegger et al. 1999). Figure 4 shows the result displayed as a histogram, with the error ratio for the original series shown as a dark line. None of the original time series appears to benefit from nonlinear forecasting. The histogram on the bottom-right of the figure shows that the time series is better predicted by the linear method. This is a result of the lower average correlation among the surrogates for this time series, shown in Figure 3.

Finally, we compare the original and surrogate time series using a time reversal asymmetry statistic. Asymmetry of the time series when reversed in time can be a signature of nonlinearity (Theiler et al. 1992). A measure of time reversibility is the ratio of the mean cubed to the mean squared differences,

(1) $$ \mathcal{Q} = \frac{E[\!(y_{i+1}-y_{i})^{3}]}{E[\!(y_{i+1}-y_{i})^{2}]}  $$

**Figure 5:**
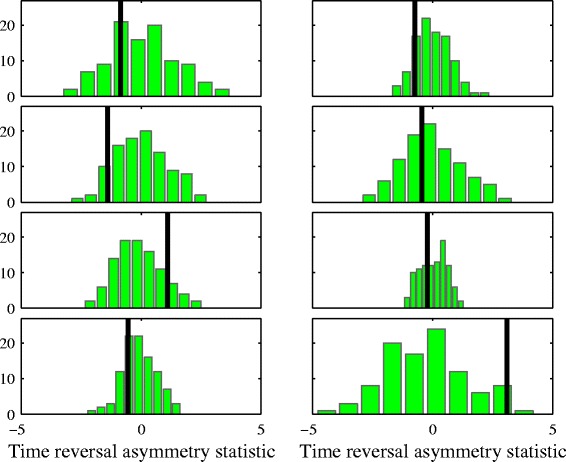
**Time reversal asymmetry statistic.** The statistic for the original time series is represented as a vertical line and that for the surrogates is shown as a histogram. Again, there is no evidence that the original time series are unusual among their surrogates except for the bottom right panel.

Figure 5 shows the  statistic values for the surrogates shown as a histogram and the statistic for the original series shown as a dark line. There is no general evidence of time asymmetry in the patients’ time series, again with the exception of the series shown on the bottom right.

### Forecasting

In this section, we apply both linear and nonlinear forecast methods to the data in order to compare the accuracy of different methods. In this way, we aim to gain some insight into the dynamics of the generating process and to evaluate the forecast methods for this application. We apply several different linear and nonlinear forecasting methods, whose details are given in Additional file 1: Section II.

Table 3 shows the out-of-sample forecast results using linear and nonlinear time series methods. The methods are persistence *PST*, simple exponential smoothing *SES*, autoregression *AR1* and *AR2*, Gaussian process regression *MAT2*, locally constant prediction *LCP* and local linear prediction *LLP*. There is little difference in accuracy between the forecasting methods. The range in error between the most and least accurate methods is less than 0.5 of a rating unit. A Deibold-Mariono test (Diebold and Mariano 2002) shows that for half the patients, none of the methods, including nonlinear methods, has more predictive accuracy than persistence forecasting. Full details of the test and results are given in Additional file 1: Section III.

**Table 3:** **Out-of-sample forecast error (RMSE) for each of the eight time series used in the study**

**Time series**	**PST**	**SES**	**AR1**	**AR2**	**MAT2**	**LCP**	**LLP**
1	3.51	2.68	2.63	2.66	2.70	2.62	2.81
2	2.28	2.08	1.88	1.87	1.83	1.85	1.91
3	4.12	4.11	3.52	3.55	3.68	3.74	3.62
4	5.08	6.56	5.15	5.24	4.88	5.51	5.09
5	2.77	2.23	2.18	2.21	2.17	2.12	2.26
6	2.61	2.61	2.49	2.41	2.45	2.89	2.43
7	3.27	2.71	2.72	2.63	2.65	2.71	2.70
8	1.59	1.59	1.53	1.59	1.60	1.70	1.53
Mean	3.16	3.07	2.76	2.77	2.75	2.89	2.79
Median	3.02	2.65	2.56	2.52	2.55	2.67	2.57

## Discussion

These results show that the eight depression time series used in this study cannot be distinguished from their linear surrogates using nonlinear and linear in-sample forecasting methods. This result contrasts with the claim in Bonsall et al. (2012) that weekly time series from patients with bipolar disorder are described better by nonlinear than linear processes. Could the divergence between the studies be a result of selection: that is, that the Bonsall et al. study tended to select nonlinear series while this study selected linear series? An earlier paper (Moore et al. 2012) reported the prediction error for 100 patients from the same monitoring scheme used by both Bonsall et al. and this study. For 100 patients, the interquartile range of prediction errors (SES) is between 2 and 4 in units of the QIDS rating scale. It can be seen that the most of the results in Table 3 lie within this range. Further, the median RMSE forecast value over 100 patients is 2.7 (0.1 normalised) and the median error in Table 3 is 2.65 (0.1). The median forecast errors in Bonsall et al. are reported as 5.7 (0.21) for the stable group and 4.1 (0.15) for the unstable group (Bonsall et al. 2012, Data supplement). We note that the data set used in the present study might not be directly comparable with that used by Bonsall et al.: for example, the time series lengths are unlikely to be the same in each set. However, for the reasons given earlier in this paper, we suggest that high prediction errors in Bonsall et al. arise from the analysis rather than the selection of time series.

The question remains as to what kind of stochastic process best describes the weekly data. The relatively better performance of the linear methods suggests a low-order autoregressive process or a random walk plus noise model (Chatfield 2002, S2.5.5), for which simple exponential smoothing is optimal (Chatfield 2002, S4.3.1). However, the identification of system dynamics, which might be high dimensional and include unobserved environmental influences would be difficult using the data available.

### Limitations

The sample of 8 patients is small in comparison with the starting set of 93 patients. The reason for the small sample size is that patients must return at least 100 ratings with fewer than 5 missing values, and the time series must be stationary: these constraints cannot easily be relaxed without compromising the analysis. However, the small sample limits how far any general inferences about dynamics of mood in bipolar disorder. Another limitation is the use of weekly data: if mood is varying over a period of days, then information about the mood dynamics is lost. Since mood telemonitoring is a relatively new technique, which relies on action by the patient, there may also be issues relating to missing or lost data. For example, Moore et al. (2013) found that uniformity of response is negatively correlated with the standard deviation of sleep ratings. This finding reveals a potential selection bias in the current study because the eight patients are selected for having fewer than five missing values, which implies a high uniformity of response. So the selected patients will all have a relatively low standard deviation of sleep ratings compared with a larger sample, but the effect, if any, on the results is unknown. Finally, the lack of control data on individuals without bipolar disorder does mean that results cannot be used to find distinguishing features of mood in the disorder.

## Conclusions

We have found that the depression time series cannot be distinguished from their surrogates generated from a linear process when comparing the respective test statistics. These results can mean that either (1) the original series have linear dynamics, (2) the test statistics for distinguishing linear from nonlinear behaviour do not have the power to detect the kind of nonlinearity present or (3) the process is nonlinear but the sampling is inadequate or the time series are too short to represent the dynamics. It is uncertain which hypothesis is to be preferred, but it would be worthwhile repeating the analysis on longer, more frequently sampled data. We note that the sample of eight patients is small, which limits how far any general inferences about dynamics of mood in bipolar disorder. On the current evidence, though, there is no reason to claim for any nonlinearity in mood time series that we examined.

## Endnote

^a^ These patients were separate from those discussed in this study.

## Additional file

Additional file 1 **Electronic supplementary material.** PDF file providing details of **(I)** data selection procedure, **(II)** forecasting methods and **(III)** Diebold-Mariono test results.
