# Supplementary material for: Mood dynamics in bipolar disorder
Source: Int J Bipolar Disord. 2014 Sep 3;2:11. doi: 10.1186/s40345-014-0011-z (PMC4452636; doi:10.1186/s40345-014-0011-z)
Supplement: Supplementary file 1 — Electronic supplementary material. PDF file providing details of (I) data selection procedure, (II) forecasting methods and (III) Diebold-Mariono test results. [file 40345_2014_11_MOESM1_ESM.pdf]

Mood Dynamics in Bipolar Disorder Electronic supplementary material

Paul J. Moore*, Max A. Little, Patrick E. McSharry, Guy M Goodwin, John R Geddes

I. DATA SELECTION

The process of data selection has three stages.

1) An initial set of 153 patients is first cleaned by removing repeated response values, that is those which share the same time stamp. These repeats arise when a patient resubmits a rating score either by mistake or in order to correct an earlier response. Assuming that earlier values are being corrected, we remove repeated responses by taking the most recent in the sequence. We then select those members whose time series have at least 25 data points, or approximately six months duration to create Set A (n=93). We resample the time series in Set A to an exact weekly sampling interval. Figure 1 illustrates the resampling process assuming that sampling is approximately once per week and that responses are valid for the previous week. The optimal weekday w for the resampled time series is chosen to minimise the total deviation of the original responses from their corresponding resampled position on the X-axis or 'comb' of weekdays. The deviation in this case is the elapsed time to the first response within seven days.

Fig. 1. Illustration of resampling. Diamond markers represent the original, non-uniform time series and the horizontal lines to the left of each marker show the period over which the response is valid. Square markers represent the resampled series and those with a square central dot are imputed values. The X-axis or 'comb' shows the optimal weekday which when aligned with the original series gives the minimum total distance (deviation) of the sample time from the response time.

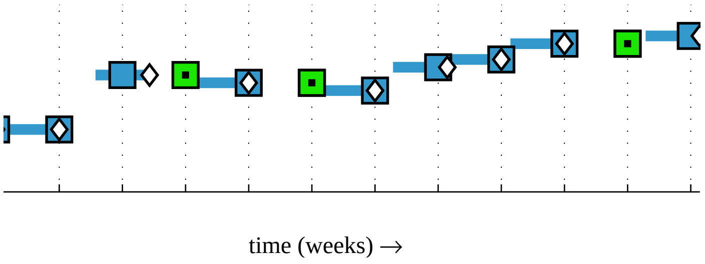

The comb is then populated from the original series as follows. Starting from weekday w at the start, or the last instance before the start, of the time series, we record any response within seven days. We repeat the search from weekday w in the following week and continue until the last response of the time series is reached. If no response is found within seven days, a missing value is imputed by using the last value in the resampled series.

2) The next step is to select those time series with a minimum resampled length of 100 data points and with 5 or fewer imputations in the first 100 points of the series. The minimum length criterion is

P. J. Moore (*Corresponding author*) is with the Mathematical Institute, University of Oxford.
M. A. Little is with the MIT Media Lab and Aston University, Birmingham UK.
P. E. McSharry is with the Smith School of Enterprise and the Environment, University of Oxford.
G. M. Goodwin is with the Department of Psychiatry, University of Oxford.
J. R. Geddes is with the Department of Psychiatry, University of Oxford.

required by the nonlinear forecasting methods and the limit on imputations avoids large errors resulting from incorrect estimation.

3) Finally, we limit the length of the time series to 100 points and remove non-stationary series to leave the 8 time series used in the study. Stationarity is tested using a Kolmogorov-Smirnov test on the first and second halfs of the time series and rejecting those found to be different at a significance level of 5%. Figure 2 summarizes the data selection process.

Fig. 2. From an initial set of 153 patients, with one time series per patient, uniform time series of length at 25 weeks are selected to give a set of 93. Next, those time series with 5 or fewer missing points in the first 100 points of the time series are selected making a set of 14. Finally, non-sta

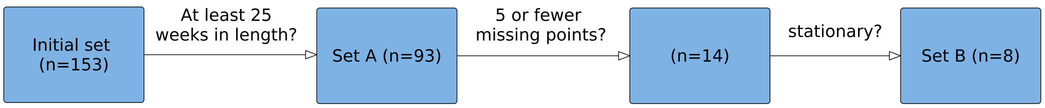

II. Forecasting methods

This section provides details of the forecasting methods used in the study. The methods are persistence *PST*, simple exponential smoothing *SES*, autoregression *AR1* and *AR2*, Gaussian process regression *MAT2*, locally constant prediction *LCP* and local linear prediction *LLP*.

A. Simple linear methods

We apply three simple linear prediction methods: *persistence*, the *autoregressive model* with orders 1 and 2, and *simple exponential smoothing*. An AR model can be written

$$y_t = \sum_{i=1}^{p} \alpha_i y_{t-i} + z_t \tag{1}$$

where $\{z_t\}$ is purely random process and p is the order of the model, which is referred to as an *AR(p)* model.

Simple exponential smoothing takes a forecast $\hat{y}_t$ at time t and adjusts it to give a next step forecast of $\hat{y}_{t+1} = \hat{y}_t + \alpha(y_t - \hat{y}_t)$, where y_t is the actual value at time t and α is a constant parameter with value between zero and one. It is estimated by taking the value of α that minimizes the root mean square error (*RMSE*) on the training data.

B. Nonlinear methods

We apply two nonlinear models, the first being a locally constant predictor, the second a local linear fit. The locally constant predictor which takes the average of the 'successor' points to the neighboring delay vectors formed from the time series. That is,

$$\hat{y}_{t+1} = \frac{1}{|\mathcal{U}(\mathbf{s})|} \sum_{s_n \in \mathcal{U}(\mathbf{s})} s_{n+1} \tag{2}$$

where $\mathcal{U}$ represents the neighborhood set of delay vectors, $\mathbf{s} = (y_{t-m+1}, \dots y_t)$ and m is the embedding dimension. The algorithm is described in [1] and is implemented using the *TISEAN* function `lzo-run` [2] with the default options. The local linear model is an extension, which uses an autoregressive model in the embedding space to form a new local model for every prediction. It is implemented by using the *TISEAN* function `lfo-run`.

C. Gaussian process forecasting

The theory for Gaussian process models is well-established and in recent decades it has been widely applied in regression and machine learning. A clear introduction to the method is to be found in [3] and a fuller description is given in the machine learning text [4]. The present authors have applied the method to forecasting in [5]. The method assumes a Bayesian nonparametric model where the regression function itself has a prior distribution. This is a Gaussian process which is specified by a covariance function $k(\mathbf{x}, \mathbf{x}' | \boldsymbol{\theta})$ to define the correlation between latent function values at inputs $\mathbf{x}$ and $\mathbf{x}'$. Properties of the prior process, such as the length scale, are determined by *hyperparameters* $\boldsymbol{\theta}$, which are estimated from the data by maximum likelihood along with a noise term σ_n. The predictive equation is then $\mathbb{E}[f_*] = \mathbf{k}_*^{\mathbf{T}}(K + \sigma_n^2 I)^{-1}\mathbf{y}$ where $\mathbf{k}_*$ is the vector of covariances of the test point with the training points $\mathbf{y}$, and K is the covariance matrix of the training set.

Fig. 3. Sample draws from a Gaussian process. The upper two plots are realizations of a Gaussian process with a Matérn covariance function $k(r) = \sigma^2 \left(1 + \frac{\sqrt{3}\,r}{l}\right) \exp\left(-\frac{\sqrt{3}\,r}{l}\right)$ with length scale $l = 10$ and standard deviation $\sigma = 5$. The two lower time plots are moving-average smoothed series selected from the eight depression time series used in ths study.

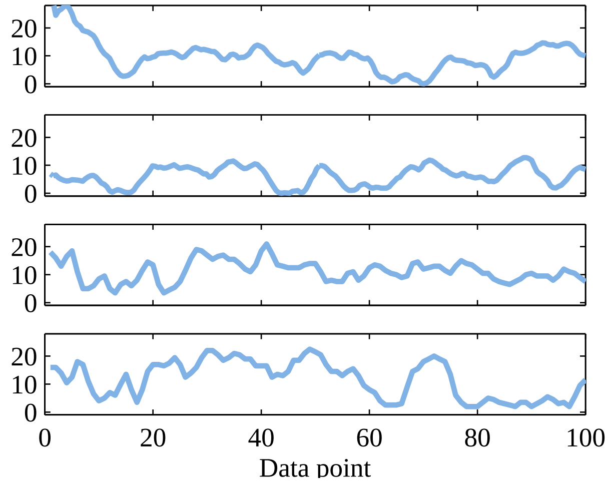

An example of draws from a Gaussian process distribution is shown in the two upper panels of Figure 3. They are generated artificially using the method described in [3]. By way of comparison, the two lower plots in Figure 3 are two moving-average smoothed time series selected from the eight used in this study. The artificial series match the smoothed mood series for time scale and variance because these hyperparameters are chosen accordingly. In forecasting, the training set is used to determine the hyperparameter values, which are then used in the predictive equation.

Choice of Gaussian process covariance function: Table I shows the negative log likelihood from training the first half of each of the eight time series used in the study. Three kinds of covariance function are used, squared exponential (*SQE*), rational quadratic (*RQU*) and Matérn (*MAT*). For the first two covariance functions and for *MAT1*, we use exact inference with a Gaussian likelihood function. For *MAT2* we use a $sech^2$ likelihood with a Laplace approximation to the posterior. The *MAT3* method uses a $sech^2$ likelihood with a leave out one inference. *MAT4* uses a t-distribution likelihood with variational Bayes inference. Further details of the inference methods can be found in [4].

The *MAT3* method is distinguished by having the lowest negative log-likelihood, but otherwise there is little to choose between the methods.

Table II shows the out of sample RMSE forecast error for the different covariance functions and inference methods. Again the *MAT3* method is distinguished from the others, but in this case because it is markedly worse. There is a large error for the time series with index 1, which was found to be random. It appears that the leave-out-one method for estimating parameters is overfitting in this case. There is little to distinguish the other methods: *MAT2* has the lowest mean out-of-sample error, but by a very small

TABLE I
NEGATIVE LOG LIKELIHOOD FOR DIFFERENT COVARIANCE FUNCTIONS, LIKELIHOODS AND INFERENCE METHODS

	SQE	*RQU*	*MAT1*	*MAT2*	*MAT3*	*MAT4*
	Gauss	*Gauss*	*Gauss*	$sech^2$	$sech^2$	*t*
Time series	*Exact*	*Exact*	*Exact*	*Lpce*	*LOO*	*VB*
1	121.0	121.0	121.0	116.9	118.2	116.9
2	122.0	122.4	122.3	122.0	119.9	124.3
3	129.6	129.0	129.5	130.8	122.6	130.4
4	156.2	156.4	156.4	156.9	152.6	181.1
5	95.3	95.6	95.4	96.5	86.5	100.4
6	107.4	107.9	107.8	110.7	98.0	109.7
7	111.6	111.9	112.2	113.1	108.0	112.7
8	122.0	120.9	120.4	120.9	98.8	121.8
Mean	120.6	120.6	120.6	120.975	113.0	124.7

TABLE II
OUT OF SAMPLE FORECAST ERROR (RMSE) FOR DIFFERENT COVARIANCE FUNCTIONS, LIKELIHOODS AND INFERENCE METHODS

	SQE	*RQU*	*MAT1*	*MAT2*	*MAT3*	*MAT4*
	Gauss	*Gauss*	*Gauss*	$sech^2$	$sech^2$	*t*
Time series	*Exact*	*Exact*	*Exact*	*Lpce*	*LOO*	*VB*
1	2.70	2.70	2.70	2.70	4.32	2.67
2	1.91	1.89	1.87	1.83	1.86	1.82
3	3.52	3.64	3.58	3.68	3.67	3.99
4	5.09	5.08	5.06	4.88	4.90	5.11
5	2.48	2.38	2.35	2.17	3.46	2.21
6	2.42	2.46	2.41	2.45	2.39	2.39
7	2.69	2.70	2.71	2.65	2.85	2.65
8	1.56	1.73	1.60	1.60	1.82	1.59
Mean	2.80	2.82	2.79	2.75	3.16	2.80

margin. For the comparison with other forecast methods, therefore, we choose *MAT2*, which uses a Matérn covariance function with a $sech^2$ likelihood and parameter inference using a Laplace approximation to the posterior.

Fig. 4. Gaussian process forecasting using a Matérn covariance function. The hyperparameters are trained on the first 50 data points to give a length scale $l = 2.5$ and a process standard deviation of $\sigma = 3.3$. The original time series, the third out of the eight used in the study, is shown as the thicker line and the prediction as the thinner line.

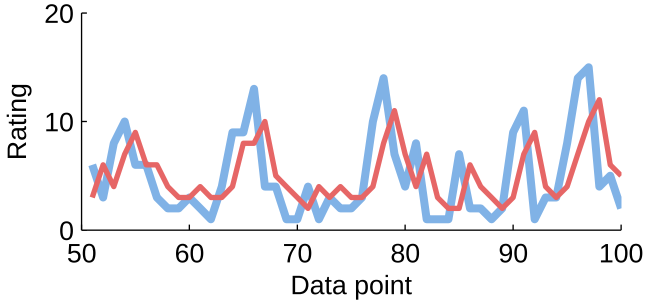

Figure 4 illustrates the forecasting method using a single time series selected from the eight used in the

study. The hyperparameters are trained on the first 50 points of the time series and next step predictions made for the next 50 points. It is possible to retrain the hyperparameters using a longer training set as the prediction point moves forward in time, but we find that this makes no difference to the prediction accuracy.

III. DIEBOLD-MARIANO TEST

The Diebold-Mariano test [6] compares the predictive accuracy of two forecasting methods by examining the forecast errors from each model. The null hypothesis of the test is that the expected values of the loss functions are the same,

$$H_0 : E[L(\epsilon_1)] = E[L(\epsilon_2)] \tag{3}$$

where ϵ_1 and ϵ_2 are the forecast errors for each method. The Diebold-Mariano test statistic for one step ahead predictions is

$$S_{DM} = \frac{\bar{d}}{\sqrt{\frac{var(d)}{T}}} \sim \mathcal{N}(0,1) \tag{4}$$

where d is $L(\epsilon_1) - L(\epsilon_2)$ and T is the number of forecasts. Since the statistic is distributed normally, we reject the null hypothesis (that the methods have equal predictive accuracy) for absolute values of above 1.96.

TABLE III
DIEBOLD-MARIANO TEST STATISTIC FOR OUT OF SAMPLE FORECAST RESULTS

Time series	*SES*	*AR1*	*AR2*	*MAT2*	*LCP*	*LLP*
1	1.98	2.21	1.98	1.90	2.06	1.64
2	0.67	1.54	1.51	1.74	1.61	1.40
3	0.02	1.83	1.55	1.71	0.74	1.38
4	-1.84	-0.12	-0.29	0.48	-0.74	-0.03
5	1.83	2.05	2.02	1.78	1.84	1.91
6	0	0.64	0.84	0.87	-0.99	0.87
7	2.25	2.87	2.67	2.49	2.06	2.31
8	0	2.15	0.16	-0.57	-0.63	0.85

Table III shows the Diebold-Mariano test statistic for out of sample forecast results used in the paper and applying an identity loss function. Forecast methods are compared individually with persistence as the baseline forecast. It can be seen that simple exponential smoothing (SES) is not distinguished from the baseline with the exception of patients 1 and 7. Patients 2,3,4 and 6 show no distinction from persistence forecasts for any of the forecasting methods, including the non-linear methods.

REFERENCES

[1] Kantz, H., Schreiber, T.: Nonlinear Time Series Analysis, 1st edn. Cambridge University Press, ??? (2003)
[2] Hegger, R., Kantz, H., Schreiber, T.: Practical implementation of nonlinear time series methods: The TISEAN package. Chaos: An Interdisciplinary Journal of Nonlinear Science **9**(2), 413–435 (1999)
[3] Rasmussen, C.E.: Gaussian processes in machine learning. In: Advanced Lectures on Machine Learning, pp. 63–71. Springer, ??? (2004)
[4] Rasmussen, C.E., Williams, C.K.I.: Gaussian Processes for Machine Learning. The MIT Press, ??? (2006)
[5] Moore, P.J., Little, M.A., McSharry, P.E., Geddes, J.R., Goodwin, G.M.: Forecasting depression in bipolar disorder. IEEE Transactions on Biomedical Engineering **59**(10) (2012)
[6] Diebold, F.X., Mariano, R.S.: Comparing predictive accuracy. Journal of Business & Economic Statistics **20**(1) (2002)
